# Effects of Switching from Degludec to Glargine U300 in Patients with Insulin-Dependent Type 1 Diabetes: A Retrospective Study

**DOI:** 10.3390/medicina60030450

**Published:** 2024-03-08

**Authors:** Toshitaka Sawamura, Shigehiro Karashima, Azusa Ohbatake, Takuya Higashitani, Ai Ohmori, Kei Sawada, Rika Yamamoto, Mitsuhiro Kometani, Yuko Katsuda, Takashi Yoneda

**Affiliations:** 1 Department of Internal Medicine, Asanogawa General Hospital, 83 Kosakamachi, Kanazawa 910-8621, Japan; sawaa4211@gmail.com (T.S.); cats2ai@yahoo.co.jp (A.O.); iekadawas0212@yahoo.co.jp (K.S.); kpmmsk@yahoo.co.jp (R.Y.); kometankomekome@yahoo.co.jp (M.K.); 2 Department of Endocrinology and Metabolism, Kanazawa University Graduate School of Medicine, 13-1 Takaramachi, Kanazawa 920-8641, Japan; azusa_k_23@yahoo.co.jp (A.O.); popfunfun@yahoo.co.jp (T.H.); yukatsudayk203@yahoo.co.jp (Y.K.); endocrin@med.kanazawa-u.ac.jp (T.Y.); 3 Department of Diabetes and Endocrinology and Internal Medicine, Fukui Prefectural Hospital, 2-8-1 Yotsui, Fukui 910-8526, Japan; 4 Department of Health Promotion and Medicine of the Future, Kanazawa University, 13-1 Takaramachi, Kanazawa 920-8641, Japan

**Keywords:** degludec, glargine U300, glycemic variability, hypoglycemia, type 1 diabetes

## Abstract

*Background and Objectives:* Degludec (Deg) and glargine U300 (Gla-300) are insulin analogs with longer and smoother pharmacodynamic action than glargine U100 (Gla-100), a long-acting insulin that has been widely used for many years in type 1 and type 2 diabetes. Both improve glycemic variability (GV) and the frequency of hypoglycemia, unlike Gla-100. However, it is unclear which insulin analog affects GV and hypoglycemia better in patients with insulin-dependent type 1 diabetes. We evaluated the effects of switching from Deg to Gla-300 on the day-to-day GV and the frequency of hypoglycemia in patients with insulin-dependent type 1 diabetes treated with Deg-containing basal-bolus insulin therapy (BBT). *Materials and Methods:* We conducted a retrospective study on 24 patients with insulin-dependent type 1 diabetes whose treatment was switched from Deg-containing BBT to Gla-300-containing BBT. We evaluated the day-to-day GV measured as the standard deviation of fasting blood glucose levels (SD-FBG) calculated by the self-monitoring of blood glucose records, the frequency of hypoglycemia (total, severe, and nocturnal), and blood glucose levels measured as fasting plasma glucose (FPG) levels and hemoglobin A1c (HbA1c). *Results:* The characteristics of the patients included in the analysis with high SD-FBG had frequent hypoglycemic events, despite the use of Deg-containing BBT. For this population, SD-FBG and the frequency of nocturnal hypoglycemia decreased after the switch from Deg to Gla-300. Despite the decrease in the frequency of nocturnal hypoglycemia, the FPG and HbA1c did not worsen by the switch. The change in the SD-FBG had a negative correlation with the SD-FBG at baseline and a positive correlation with serum albumin levels. *Conclusions:* Switching from Deg to Gla-300 improved the SD-FBG and decreased the frequency of nocturnal hypoglycemia in insulin-dependent type 1 diabetes treated with Deg-containing BBT, especially in cases with low serum albumin levels and a high GV.

## 1. Introduction

The goal of diabetes treatment is to prevent diabetic complications and achieve a long healthy life span. Strict blood glucose control is important to prevent micro- and macro-vascular diabetic complications in patients with diabetes [1,2] However, the Action to Control Cardiovascular Risk in Diabetes (ACCORD) trial, in which the efficiency of the normalization of blood glucose levels via insulin or sulfonylurea was evaluated, revealed that mortality increased through the normalization of hemoglobin A1c (HbA1c) levels via insulin or sulfonylurea [3]. The additional analysis of the ACCORD study revealed that symptomatic, severe hypoglycemia was associated with an increased risk of death [4]. Following this report, more attention has been paid to hypoglycemia. Various studies have shown that overly intensive control induces hypoglycemia and increases the risk of cardiovascular events and mortality [5,6]. Moreover, it has also been noted that hypoglycemia increases the incidence of dementia [7] and the rate of bone fractures [8]. For this reason, avoiding hypoglycemia is one of the most important tasks in diabetes care. In addition to hypoglycemia, the concept of glycemic variability (GV) as a quality of glycemic control has also received attention. GV encompasses various types of variability, including diurnal variability, day-to-day variation, and even seasonal variability. All of these have been reported to correlate with diabetic complications [9]. Among several GV evaluations, the day-to-day GV is a marker that reflects short-to-intermediate GV. A high day-to-day GV is reportedly related to diabetic complications [10,11]. This is explained by the mechanism of exacerbation of oxidative stress due to high day-to-day GV [12]. Moreover, patients with high GV often experience hypoglycemia [13], and hypoglycemia itself could increase the risk of cardiovascular events and mortality [4,5,6]. Sakamoto et al. reported the factors affecting GV. In the short-term and intermediate GV, beta-cell dysfunction, genetic factors, and insulin resistance have strong contributions. Habitual practice has a major impact on long-term GV. Diet, activity, stress, the effect of medications, and adherence to medications affect all types of GV [9].

As severe beta-cell dysfunction leads to a worsening short-to-intermediate GV, patients with insulin-dependent type 1 diabetes have high day-to-day GV and experience more frequent hypoglycemic events compared with insulin-independent type 2 diabetes [14]. For patients with insulin-dependent type 1 diabetes, subcutaneous multiple insulin injection therapy often used. However, some patients suffer from high day-to-day GV and hypoglycemia, despite the use of subcutaneous multiple insulin injection therapy. Recently, a hybrid closed-loop insulin delivery system could be available in type 1 diabetes. This system uses various combinations of control algorithms, glucose sensors, and insulin pumps. A hybrid closed-loop insulin delivery system could achieve increased time in target, and reductions in HbA1c, hyperglycemia, and hypoglycemia, compared to that with an insulin pump [15]. However, there are some barriers to the use of a hybrid closed-loop insulin delivery system, such as the high financial burden and the difficulty of using it with elderly people and patients with dementia due to the need to handle the machine. Therefore, many patients with insulin-dependent type 1 diabetes are treated with subcutaneous multiple insulin injection therapy.

For the stabilization of blood glucose levels in patients with insulin-dependent type 1 diabetes treated with basal-bolus insulin therapy (BBT), basal insulin has an important role. Insulin glargine U100 (Gla-100) is a long-acting insulin widely used in both type 1 and type 2 diabetes. However, insulin glargine U300 (Gla-300) and insulin degludec (Deg), which have prolonged pharmacodynamic action, can be used recently. Gla-300 achieves a smoother day-to-day GV and a decreased frequency of hypoglycemia compared with Gla-100 in patients with type 1 [16,17] and type 2 [18,19] diabetes. Similarly, Deg also achieves a smoother day-to-day GV and decreases the frequency of hypoglycemia compared with Gla-100 for patients with type 1 [20,21] and type 2 [22,23] diabetes. As Deg has a longer pharmacodynamic action profile than Gla-300, Deg tends to be more commonly used for patients with type 1 diabetes. However, a high day-to-day GV and frequent hypoglycemia in type 1 diabetes can often be observed, despite the use of Deg [14]. 

The previous reports on type 2 diabetes have shown that Gla-300 can decrease the frequency of hypoglycemia compared with Deg [24,25]. Especially, in patients with low serum albumin levels, Gla-300 could achieve a lower GV and decreased frequency of hypoglycemia than Deg. However, the data about the comparison between these two insulin analogs in type 1 diabetes is limited. We hypothesized that switching from Deg to Gla-300 may improve GV and decrease hypoglycemic events in patients with type 1 diabetes and that there is a group of patients who benefit more from Gla-300 than Deg. The standard deviation of fasting blood glucose levels (SD-FBG), calculated by the self-monitoring of blood glucose (SMBG) records, is used as the marker of day-to-day GV [26]. Here, we evaluated the efficiency of switching from Deg to Gla-300 on the SD-FBG calculated by the SMBG records and the frequency of hypoglycemic events in patients with insulin-dependent type 1 diabetes. 

## 2. Materials and Methods

### 2.1. Patients

We investigated the outpatients who attended the Endocrinology and Diabetic unit of Fukui Prefectural Hospital and Asanogawa General Hospital from April 2017 to December 2022. The eligible patients were male and female patients including the following: (1) patients with insulin-dependent type 1 diabetes, (2) patients who had been treated with Deg-containing BBT and Deg was switched to Gla-300 for various reasons, and (3) patients who had been instructed to perform SMBG four times/day. Among these patients, we excluded patients as follows: (1) patients who had changed antidiabetic agents or received new nutritional guidance during the observation period, (2) patients who were newly introduced to flash glucose monitoring (FGM) during the observation period, and (3) patients whose HbA1c levels and SMBG records could not be obtained within 1 month and 4 to 6 months after the insulin switch.

### 2.2. Measurement

The primary endpoint of this study is the change in SD-FBG calculated by SMBG records. SD-FBG was calculated from the 30 records measured before breakfast. The secondary endpoints are the change in the following items: body weight (BW), body mass index (BMI), fasting plasma glucose (FPG), HbA1c, serum creatinine (Cr), estimated glomerular filtration rate (eGFR), serum albumin (Alb), frequency of hypoglycemia, frequency of severe hypoglycemia, frequency of nocturnal hypoglycemia, and a dosage of basal and fasting insulins. Moreover, time above range (TAR) (>180 mg/dL), time in range (TIR) (70 to 180 mg/dL), and time below range (TBL) (<70 mg/dL) were evaluated only in patients with FGM.

The first evaluation points were within 1 month of the insulin switch, and the second evaluation points were 4 to 6 months after the insulin switch. SD-FBG was calculated from the records of SMBG in the previous 30 times. Hypoglycemia is defined as a blood glucose level below 70 mg/dL or having hypoglycemic symptoms. Severe hypoglycemia is defined as a blood glucose level below 54 mg/dL or hypoglycemia that requires treatment assistance from another person. Nocturnal hypoglycemia was defined as hypoglycemia occurring from 00:00 h until the next breakfast. 

### 2.3. Ethics Conduct

This study utilized a retrospective design and was approved by the ethics committee at Fukui Prefectural Hospital (No. 18-69) and Asanogawa General Hospital (No. 216) with a waiver of consent obtained from the committee. All procedures were performed following the 1964 Helsinki Declaration and its later amendments.

### 2.4. Statistical Analysis 

The data are expressed as mean ± SD and were analyzed using the statistical software package EZR version 1.55 (Saitama Medical Center, Jichi Medical University, Saitama, Japan), which is a graphical interface for R (The R Foundation for Statistical Computing, Vienna, Austria) [27]. *p*-values < 0.05 indicated statistical significance. For the comparisons of the variables, a pairwise *t*-test was used for normally distributed data, and a Wilcoxson test was used for non-normally distributed data. A correlation analysis was performed using the Pearson test to validate the correlation factors affecting the change in the SD-FBG. No statistical sample size calculations were conducted, as this study is a retrospective design.

## 3. Results

A total of 27 patients with insulin-dependent type 1 diabetes were switched from Deg-containing BBT to Gla-300-containing BBT from April 2017 to December 2022. Three patients were excluded from the analysis for the following reasons: (1) newly introduced to FGM during the observation period (two patients), and (2) HbA1c level and SMBG records could not be obtained at 4 to 6 months after the insulin switch (one patient). Another 24 patients with insulin-dependent type 1 diabetes whose treatment was switched from Deg-containing BBT to Gla-300-containing BBT were retrospectively analyzed. The clinical characteristics are summarized in Table 1. The patients had a mean age of 56.0 ± 15.2 years, a mean diabetes duration of 14.1 ± 13.6 years, and the percentage of females was 46% (11/24). At the baseline evaluation, the mean levels of HbA1c were 7.8 ± 0.6%, and the mean BMI was 22.1 ± 2.7 kg/m^2^, respectively. The mean dosage of fasting insulin and basal insulin were 0.38 ± 0.14 units/kg, and 0.20 ± 0.10 units/kg, respectively. The mean SD-FBG was high, at 58.2 ± 18.2 mg/dL, and the average counts of total hypoglycemia and severe hypoglycemia per month were 7.0 ± 5.6 times/month and 1.0 ± 1.3 times/month, respectively. The percentage of patients with FGM was 58% (14/24).

The parameters before and after the insulin switch are also shown in Table 1. The SD-FBG significantly decreased after the switch from Deg to Gla-300 (baseline: 58.2 ± 18.2 mg/dL, after the switch: 49.7 ± 15.7 mg/dL, *p* = 0.02). Moreover, the frequency of nocturnal hypoglycemic events decreased after the insulin switch (baseline: 2.5 ± 2.1 times/month, after the switch: 1.5 ± 1.3 times/month, *p* = 0.003). The frequency of total hypoglycemic events tended to decrease after the insulin switch, but there were no statistical differences. There was no difference in the frequency of severe hypoglycemia before and after the insulin switch. Despite the decrease in nocturnal hypoglycemic events, the HbA1c levels after the insulin switch did not worsen (baseline: 7.8 ± 0.6%, after the switch: 7.7 ± 0.5%, *p* = 0.27). There were no differences in the dosage of fasting insulin (baseline: 22.2 ± 8.3 units, after the switch: 22.2 ± 8.1 units, *p* = 0.94) and basal insulin (baseline: 12.3 ± 7.1 units, after the switch: 12.7 ± 6.1 units, *p* = 0.27) before and after the insulin switch. No changes were observed in other parameters.

In the analysis of the patients with FGM, the TBL decreased after the switch from Deg to Gla-300 (baseline: 6.8 ± 3.7%, after the switch: 4.1 ± 1.6%, *p* = 0.01). Despite the decrease in the TBL, the TAL and TIR after the insulin switch did not worsen.

**Figure 1:**
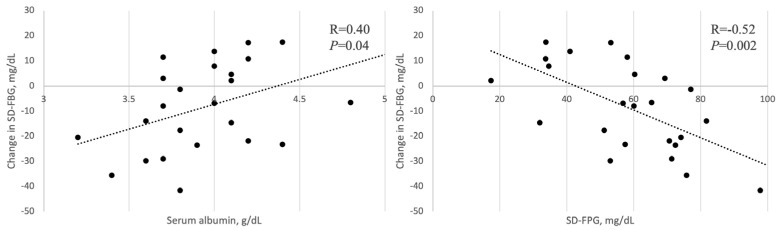
Correlation between the change in SD-FBG and Alb, SD-FBG at baseline.

We described the factors associated with the change in the SD-FBG after the switch from Deg to Gla-300 (Figure 1). The change in the SD-FBG had a negative correlation with the SD-FBG at baseline (*r* = −0.52, *p* = 0.002) and a positive correlation with the Alb (*r* = 0.40, *p* = 0.04). There were no correlations between the change in the SD-FBG and other parameters.

## 4. Discussion

Herein, we evaluate the effect of the switch from Deg to Gla-300 on the GV and the frequency of hypoglycemia in patients with insulin-dependent type 1 diabetes treated with Deg-containing BBT. In the first part of the discussion, we describe the unique baseline characteristics of the patients included in this study. In our study, the included patients had a large mean SD-FBG of 58.2 ± 18.2 mg/dL. In a previous Japanese observational study in which the SD-FBG was evaluated in patients with type 1 diabetes, the mean SD-FBG level was 47.5 ± 22.0 mg/dL [28]. In this study, the titer of insulin antibodies was comprehensively measured in patients with type 1 diabetes treated via insulin injection therapy and still had a clinically high GV. In the population who suffered from high GV, the value of the SD-FBG was lower compared with that in our study. This indicated that the population in our research had a markedly lower GV than those under the usual care in Japan. Moreover, the rate of hypoglycemic events was also high compared with that in the previous study. In the SWITCH 1 randomized clinical trial [29], in which the efficiency of Deg compared with Gla-100 was evaluated in patients with type 1 diabetes, the total, nocturnal, and severe hypoglycemia events in patients with Deg were 2.0 times/month, 0.30 times/month, and 0.07 times/month, respectively. Similarly, the BEGIN basal-bolus type 1 trial [20], which is a phase 3, randomized, open-label, treat-to-target, non-inferiority trial of Deg in patients with type 1 diabetes, the total, nocturnal, and severe hypoglycemia in patients with Deg were 3.5 times/month, 0.37 times/month, and 0.02 times/month, respectively. These hypoglycemic rates are lower than those in our study. Therefore, the patients included in our study could be rephrased as patients treated with Deg-containing BBT, but who were not well controlled in terms of GV and hypoglycemia. In this population, switching from Deg to Gla-300 improved the day-to-day GV, expressed by the SD-FBG, and decreased the frequency of nocturnal hypoglycemia. The change in the SD-FBG had a negative correlation with the SD-FBG at baseline and a positive correlation with the Alb.

Deg and Gla-300 improve day-to-day GV, unlike Gla-100, owing to their longer pharmacodynamic effect [16,17,18,19,20,21,22,23]. However, the action mechanisms of these two ultra-long-acting insulin analogs are different. Deg forms a soluble multi-hexametric chain after subcutaneous injection and the zinc moiety of the insulin molecule diffuses slowly from the terminal ends of Deg and gets absorbed into circulation. After the absorption into the circulatory system, almost all Deg binds to albumin and is slowly released from the albumin within the target tissue to achieve a hypoglycemic effect [30]. In contrast, Gla-300 does not bind to albumin when in circulation [31]. The previous report showed that Alb levels fluctuate daily, with high values in the daytime and low values at night [32]. The decrease in the Alb level increases the free insulin levels and could decrease blood glucose levels. Therefore, a high GV and frequent nocturnal hypoglycemia are thought to be improved through switching from Deg to Gla-300.

Kawaguchi et al. reported that a lower GV and a decreased frequency of hypoglycemia are observed in patients with Gla-300 compared with Deg in type 2 diabetes [25]. Their findings indicated that the frequency of nocturnal hypoglycemia in patients with Deg had an association with low serum albumin levels. However, no association was observed between serum albumin levels and the frequency of nocturnal hypoglycemia in patients with Gla-300 [25]. Another report on type 2 diabetes showed that Gla-300 decreased the total and nocturnal hypoglycemia compared with Deg in patients with Alb < 3.8 g/dL [33]. Although these reports differ in that they are based on type 2 diabetes, their results are similar to ours. However, opposite results were also reported on type 2 diabetes. Tibaldi et al. reported that the administration of Deg to patients with type 2 diabetes achieved a greater HbA1c reduction with fewer hypoglycemic events after 6 months from the administration compared with those in patients with Gla-300 [34]. Studies on type 1 diabetes are limited and the results are also controversial. A double-blind crossover euglycemic clump study showed that Gla-300 induced 20% less fluctuation in steady-state glucose infusion rate profiles than that of Deg in a once-daily morning dosing regimen of 0.4 U/kg/day [35]. However, the opposite result was obtained in another double-blind crossover euglycemic clump study [36]. 

Recently, Miura et al. conducted a multicenter crossover trial on type 1 diabetes in which the efficiency of Deg and Gla-300 on the SD-FBG were evaluated [37]. In this study, 46 patients with insulin-dependent type 1 diabetes were randomly assigned to the Deg-first/Gla-300-s group or the Gla-300-first/Deg-second group and treated with the respective basal insulin for 4-week periods. The primary endpoint of this study was to examine the noninferiority of Deg compared to Gla-300 regarding day-to-day GV evaluated as SD-FBG levels by the SMBG records. This study indicated that the SD-FBG during the Deg treatment period was not inferior to that during the Gla-300 treatment period (mean difference of −6.6 mg/dL, with a 95% CI of −16.1 to 3.0 mg/dL). Among 46 patients included in this study, 32 patients were evaluated using continuous glucose monitoring. In these 32 patients, the TBLs (<70 mg/dL) were shorter during the Gla-300 treatment period, and the TALs (>180 mg/dL) were shorter during the Deg treatment period, respectively. In their conclusion, they identified that these two insulins have comparable glucose-stabilizing effects in patients with insulin-dependent type 1 diabetes. However, there are cases of insulin-dependent type 1 diabetes in which there is a large clinical difference in efficacy between Deg and Gla-300. 

There are several important differences between this crossover study [37] and our study. The first was the eligible patients. In our study, patients whose treatments were switched from Deg to Gla-300 for various reasons in real-world medical examinations were included. Therefore, patients with a stable blood glucose control with Deg might not be analyzed. The patients included in our study had a high GV and frequent hypoglycemic events despite the use of Deg-containing BBT. The second difference was the length of observation. Our study has a longer follow-up period compared with the past study by Miura et al. [37]. In general, the longer the observation period, the more it is affected by factors other than simple medication-to-medication differences, including diet and exercise. A past study [37] indicated that treatment with Gla-300 achieved a lower hypoglycemic rate compared to that with Deg. However, there was no difference in the SD-FBG between treatment with Gla-300 and treatment with Deg. Hypoglycemic events could make patients feel hunger and increase appetite. Therefore, the improvement in GV in our study may be affected by the fact that Gla-300 treatment reduced the frequency of hypoglycemia and improved the hypoglycemia-induced increase in appetite. However, no evaluation of appetite and food intake was conducted in our study. The third difference was in the evaluation of factors correlating with the change in the SD-FBG. The previous study [37] did not evaluate the factors influencing the superiority of these ultra-long-acting insulin analogs. In contrast, our study showed that low serum albumin level and a high SD-FBG with Deg-containing BBT are the predictors of the superiority of Gla-300 over Deg in insulin-dependent type 1 diabetes. Knowing these predictor markers may lead to the personalization of treatment in patients with insulin-dependent type 1 diabetes. Although not considered in our current research, the longer pharmacological action of Deg compared with that of Gla-300 should be considered. During the use of Deg, the efficiency and safety of a flexible dosing regimen at fixed intervals with a minimum of 8 h and a maximum of 40 h between each injection was reported [38]. There is no report about a flexible dosing regimen used with Gla-300. However, the pharmacological action of Gla-300 is much shorter than that of Deg. Thus, it is unlikely that the results of using a flexible dosing regimen with Gla-300 will be as favorable as those with Deg. For this reason, Deg is expected to be more useful than Gla-300 in cases where insulin dosing times vary from day to day. 

Our study has several limitations. First, this study adopted a retrospective design and was conducted on a small number of patients. In general, larger sample sizes are more likely to yield significant differences when examining differences between two medications. However, even though no significant difference was found in the existing studies with large sample sizes, the present study found a significant difference in the SD-FBG. We believe that the small sample size is a limitation, but also a possible new finding that some groups may benefit from treatment modification. The eligible patients in our study were patients with insulin-dependent type 1 diabetes and whose medications were switched from Deg-containing BBT to Gla-300-containing BBT for various clinical problems and have a high GV and frequent hypoglycemia despite the use of Deg. We would like to inform readers that the switch from Deg to Gla-300 may not be effective in all patients with insulin-dependent type 1 diabetes. The results of this study could be considered applicable to patients with insulin-dependent type 1 diabetes with Deg-containing BBT but who have a high GV or frequent hypoglycemia. The sample size is smaller than existing studies, but the significant difference from Deg to Gla-300, and the fact that the population is different from previous studies, suggest that a treatment change is likely to be effective in certain groups. Therefore, it is desirable to conduct a randomized, prospective study of switching from Deg to Gla-300 in patients with insulin-dependent type 1 diabetes treated with Deg-containing BBT and having high GV or low serum albumin levels, which were shown in our observation study to benefit from the switch from Deg to Gla-300. The next limitation is that the evaluation of GV was made by the records of SMBG which is inferior to that evaluated by CGM. Therefore, it is hoped that in the next study, the assessment of GV will be done using CGM rather than SMBG.

## 5. Conclusions

Switching from Deg to Gla-300 is effective for improving day-to-day GV and decreasing nocturnal hypoglycemia in patients with insulin-dependent type 1 diabetes and having high day-to-day GV despite the use of Deg-containing BBT. The effectiveness of improving day-to-day GV is greater in cases with low serum albumin levels and large day-to-day GV despite the use of Deg-containing BBT. 

## Figures and Tables

**Table 1:** Comparison of parameters before and after the switch from Deg to Gla-300. Data are represented as mean ± SD.

Variable	Baseline	After the Switch	*p*-Value
Age, years	56.0 ± 15.2		
Male, *n* (%)	13 (54%)		
Duration of diabetes, years	14.1 ± 13.6		
BW, kg	58.2 ± 9.8	58.3 ± 9.5	0.84
BMI, kg/m^2^	22.1 ± 2.7	22.1 ± 2.5	0.80
FPG, mg/dL	135 ± 57.1	142 ± 64.4	0.68
HbA1c, %	7.8 ± 0.6	7.7 ± 0.5	0.69
Cr, mg/dL	0.92 ± 0.47	0.86 ± 0.35	0.27
eGFR, mL/min/1.73 m^2^	74.6 ± 29.7	75.0 ± 27.5	0.81
Alb, g/dL	3.9 ± 0.3	3.9 ± 0.3	0.80
Fasting insulin dosage, units	22.2 ± 8.3	22.2 ± 8.1	0.94
Basal insulin dosage, units	12.3 ± 7.1	12.7 ± 6.1	0.27
Fasting insulin dosage, units/kg	0.38 ± 0.14	0.38 ± 0.12	0.81
Basal insulin dosage, units/kg	0.20 ± 0.10	0.21 ± 0.08	0.16
SD-FBG, mg/dL	58.2 ± 18.2	49.7 ± 15.7	0.02
Frequency of total hypoglycemia, times/month	7.0 ± 5.6	6.3 ± 4.6	0.24
Frequency of severe hypoglycemia, times/month	1.0 ± 1.3	1.0 ± 1.1	0.80
Frequency of nocturnal hypoglycemia, times/month	2.5 ± 2.1	1.5 ± 1.3	0.003
TAR, % (*n* = 14)	38.3 ± 7.4	39.6 ± 11.1	0.58
TIR, % (*n* = 14)	54.9 ± 7.3	56.4 ± 10.2	0.46
TBR, % (*n* = 14)	6.8 ± 3.7	4.1 ± 1.6	0.01

BW: body weight; BMI: body mass index; FPG: fasting plasma glucose; HbA1c: Hemoglobin A1c; Cr: creatinine; eGFR: estimated glomerular filtration rate; Alb: albumin; SD-FBG: standard division.

## Data Availability

The data are available from the corresponding author upon reasonable request.
